# Comparative Study of Frequency of Alveolar Osteitis, with and without using Platelet-Rich Fibrin in Mandibular Third Molar Surgery

**DOI:** 10.1155/2023/2256113

**Published:** 2023-03-31

**Authors:** Maryam Asif, Ahsan Ullah, Hasan Mujtaba, Muhammad Farooq Umer, Zohaib Khurshid

**Affiliations:** ^1^Oral and Maxillofacial Surgery Department, Pakistan Institute of Medical Sciences, Islamabad, Pakistan; ^2^Oral and Maxillofacial Surgery Department, School of Dentistry, Shaheed Zulfiqar Ali Bhutto Medical University, PIMS G8/3, Islamabad 04485, Pakistan; ^3^Department of Oral Pathology, School of Dentistry, Shaheed Zulfiqar Ali Bhutto Medical University, PIMS G8/3, Islamabad 04485, Pakistan; ^4^Department of Preventive Dental Sciences, College of Dentistry, King Faisal University, Hofuf, Al-Ahsa 31982, Saudi Arabia; ^5^Department of Prosthodontics and Dental Implantology, King Faisal University, Hofuf, Al-Ahsa, Saudi Arabia

## Abstract

**Introduction:**

Alveolar ostitis (AO) is the dissolution of blood clot due to enhanced local fibrinolysis and is caused by trauma to the jaw (direct) or because of bacterial involvement (indirect), which result in the activation of plasminogen pathway. Platelet-rich fibrin (PRF) is a platelet concentrate that comprises numerous autologous growth factors, and immune cells hence has the potential to expedite the healing process. The objective of the study was to determine the efficacy of PRF in the surgically extracted third molar in the context of its potential progress to AO.

**Materials and Methods:**

A total of 180 patients of 18–65 years with unilateral painful mandibular third molars due to caries, failed endodontics treatment, and pericoronitis were included in the study. Exclusion criteria were patients who were medically compromised, smokers, alcoholic, poor oral hygiene, third molar having associated periapical pathology, and receiving antibiotic regime in the last 2 weeks. Before starting surgical procedure, patients were randomly divided into two groups using lottery method. Group I received PRF in the extraction socket, while in Group II, the extraction site was left for normal healing as practiced in a standard procedure. Pain was assessed in terms of pain score, and it was recorded on a 10 mm visual analog scale on the first and third postoperative days.

**Results:**

Mean age of the patients was 41.35 ± 9.87 years. The mean age in Group I was 42.84 ± 10.52 years, and in Group II, it was 40.54 ± 9.52 years. Out of 180 patients, 90 (50.0%) were male and 90 (50.0%) were female, with a male-to-female ratio of 1 : 1. Frequency of AO following mandibular third molar surgery in Group I receiving PRF was 2.22% and in non-PRF group 12.22% (*p*-value = 0.010).

**Conclusion:**

The incidence of AO following mandibular third molar surgery was lower when PRF was used.

## 1. Introduction

Dry socket, also known as alveolar ostitis (AO), is postextraction pain discomfort inside or surrounding area of tooth socket that worsens on the second or third day after the removal of tooth. It is primarily due to complete or partial loss of the blood clot due to fibrinolytic activity [1]. It is a self-limiting condition, but signs and symptoms can extend up to 28 days. Consequently, treatment should be started as soon as possible after diagnosis to speed up recovery time, minimize pain, and enhance the quality of life [2]. Depending on the extent of tissue trauma caused by tooth extraction, the incidence ranges from 1% to 5% for normal to over 30% for permanent molars extraction. Cigarette smoke and poor oral hygiene are considered the most common contributing factors. Patients with AO are more likely to pursue the treatment within a week because of severe pain, halitosis, and dysphagia [1–3]. The pathophysiology of AO is the dissolution of a blood clot due to increased local fibrinolysis. It is either because of direct (trauma to the jaw) or indirect (bacterial) factors which result in the activation of the plasminogen pathway [4].

The complexity of surgery, degree of trauma, surgeon's expertise, age, gender, tobacco smoking, mandibular third molar, use of oral contraceptive drugs, and systemic disorders are all predisposing factors for AO [5]. Local factors like increased bone density, decreased blood flow, and decreased granulation tissue formation are thought to contribute to an elevated risk of AO at the mandibular third molar extraction site [6, 7]. Systemic analgesia, antibiotics, and intraalveolar dressing are used to treat AO, although other strategies for prevention have been proposed, including systemic and topical antibiotics, chlorhexidine, steroids, polylactic acid, and tranexamic acid [8, 9].

Platelet-rich fibrin (PRF) is the second-generation platelet concentrate. It is prepared using a simple and low-cost method that does not involve the use of neurochemical blood processing [10]. PRF has a variety of uses in dental procedures, such as extraction socket preservation, periapical surgery, and augmentation in implant surgery [11]. Basically, it is a fibrin layer of connective tissue that is sutured around the wounds to cover up the exposed extraction socket. Due to its distinctive microstructure and potential usage, PRF is extremely good for enhancing tissue repair [12, 13].

Only a few studies have been undertaken internationally regarding the effectiveness of PRF for the prevention of AO after third molar extraction. The objective of the study was to compare the surgical outcome with and without PRF in the extraction socket following mandibular third molar surgery in the context of its predilection of developing AO.

## 2. Materials and Methods

This comparative study was conducted in the Department of Oral and Maxillofacial Surgery, School of Dentistry, Shaheed Zulfiqar Ali Bhutto Medical University Islamabad, from March 2022 to September 2022. Approval of the study was granted by the institutional review board (No. SOD/ERB/2022/119) before the commencement. The study protocols were explained to the participants, and privacy was ensured, followed by informed written consent from all the participants.

A total of 180 cases of both gender between 18 and 65 years presented with unilateral painful mandibular third molars due to caries, failed endodontics treatment, and pericoronitis were included. Medically compromised patients, smokers, alcoholic, third molar with associated periapical pathology, poor oral hygiene, and receiving antibiotic regimes in the last 2 weeks were excluded. A structured proforma was used to record the patient's demographic details like name, age, and gender. Data were collected by a single independent researcher to control the bias of observation. Confounding variables like age and gender were controlled by restriction of extreme age and stratifying into age groups.

Before starting surgical procedure, patients were randomly assigned to one of the groups using lottery method. Group I received PRF in the extraction socket and then closed, while Group II had the extraction socket closed using the standard procedure. Cephalic or basilica vein of the patient was pricked with a 19-gauge needle, and 10 ml of peripheral venous blood was withdrawn. The blood was instantly separated by centrifugation at 3,500 rpm for 10 min. Relative centrifugal force (RCF) was calculated using the formula RCF = (RPM)^2^ × 1.118 × 10^−5^ × *r*. A 10 ml glass tube was used, our centrifuge rotor radius was 8 cm, RPM was 3,500/min, and the *G* force for centrifuge was calculated ∼1,096 *g* [14, 15]. At the end of centrifugation, the fibrin clot (PRF) in the center of the tube was collected. Povidone-iodine was applied on the face, 2% lidocaine in 1 : 100,000 epinephrine cartridges was administered, full mucoperiosteal envelop flap was used to access the tooth for removal. Bone contouring was performed with a low-speed handpiece irrigated with normal saline. The flap was sutured with 3-0 silk sutures after PRF was placed in extraction sockets (Group I). In Group II (non-PRF), the socket was closed with silk 3-0 sutures without the use of PRF and observed for healing. For 5 days, patients were given amoxicillin 1,000 mg two times a day and naproxen 550 mg two times a day.

Pain was assessed in terms of pain score and it was recorded on 10 mm visual analog scale (VAS) on the first and third postoperative day. Postoperative blood clot and exposure of bone were evaluated clinically, and AO was labeled as per operational definition [1].

All collected data were entered into the Statistical Package for Social Sciences (SPSS) version 21.0, and the results were analyzed. The quantitative variables, such as age and pain, were presented as means with standard deviations (SD). The qualitative variables in the data, such as gender and AO, were presented as frequency and percentages. Poststratification *χ*^2^ test was applied for qualitative variables with a *p*-value of ≤0.05 considered statistically significant.

## 3. Results

The participants' age ranged from 18 to 65 years, with a mean of 41.35 ± 9.87 years.  Table 1 shows the mean age in Group I was 42.84 ± 10.52 years and in Group II was 40.54 ± 9.52 years, respectively.  Table 2 shows that out of 180 patients, 90 (50.0%) were male and 90 (50.0%) were female, with a male-to-female ratio of 1 : 1. The average VAS score was 3.22 ± 1.09, and there was no mean difference of pain in both groups. With a *p*-value of 0.010, the prevalence of AO after mandibular third molar surgery in Group I (PRF group) was 2.22 (total of two patients) and in Group II (non-PRF group) was 12.22 (nine patients), as shown in Table 3.

AO was analyzed in both groups with respect to age, and no significant difference was observed in the age group of 18–40 years (*P* = 0.142). In the age group 41–65 years, there was a statistically significant correlation between the groups (PRF and non-PRF), having a *p*-value of 0.027 (Table 4). Stratification of AO with gender was performed in both groups, and a statistically significant difference was seen in females (*p*-value = 0.024), while in males, it was not significant (Table 5).

## 4. Discussion

It is widely known that the severity of discomfort peaks around the first 24 hr of extraction. The severity of symptoms associated with AO is characterized by progressively intense, debilitating symptoms that last all night. The pain does not react well to common analgesic medications, and maximum intensity is reported 72 hr after extraction [1, 5, 7]. PRF has been found to reduce pain and inflammation after tooth extraction along with augmenting soft tissue repair [12]. PRP is the first generation, while PRF is the second generation of platelet concentrate [13, 14]. It is produced using a straightforward, low-cost method that avoids the use of biochemical blood. In oral surgery, PRF has a variety of uses, including socket preservation, endodontic surgery, and implant surgery [16–19].

In our study, the frequency of AO following mandibular third molar surgery in Group I (PRF group) was 2.22% and in Group II (with non-PRF group) was 12.22% with a *p*-value of 0.010. Similar results for AO have been reported by Hoaglin and Lines [20], reporting 1% (2 out of 200) of prevalence in PRF treated while 9.5% (19 out of 200) in non-PRF group. Dohan et al. [21] used PRF as a filling material in an extraction socket and revealed that PRF could establish neovascularization and epithelial lining of the extraction socket. Finally, in infected and inflamed wounds, rapid wound healing is envisaged with no pain, dryness, or purulent complications. This shows that using PRF as a grafting material for dry socket treatment may improve clinical recovery due to the abundance of growth factors liberated from PRF mesh, including platelet-derived growth factor, transforming growth factor beta, epidermal growth factor, insulin-like growth factor, and hepatocyte growth factor [21].

Rutkowski et al. [22] reported a significant reduction in the incidence of AO after the extraction site was treated with PRF. The hemostasis and cicatricial features of PRF may play a role in lowering the frequency of AO following PRF treatment [23]. PRF creates a three-dimensional framework that contains platelets, leucocytes, and different cytokines/fibrinogen degradation products (FDPs) that drive neutrophil migration. FDPs boost the expression of CD 11c and CD 18 receptors, which aid neutrophil motility [24]. It also forms a natural fibrin matrix that aids in the development of clots and preserves them from mechanical dislodgment. The capability of the PRF to seal the socket inhibits pathogens from entering. This efficient and cost-effective treatment can be used as a preventive strategy for dental practitioners to minimize the possibility of AO in patients [23, 24].

In our study, the incidence of AO seems to increase with age, and statistically significant correlation was observed between age and AO having a *p*-value of 0.027, thus consistent with the majority of the reported literature [25].

Kumar et al. [26] found that AO is uncommon before the age of 18 because bone marrow is hemopoietic and transforms into fat marrow after 40 years; therefore, the prevalence of fibrinolytic alveolitis is low or nonexistent due to the lack of a stable tissue activating agent. Conversely, Nusair and Younis [27] found that AO is common in patients of 18–33 years and suggested that the well-developed alveolar bone and a lower incidence of periodontal diseases might lead to difficult tooth extractions, which potentially lead to AO.

Gender and AO in our study also showed a statistically significant correlation (*p*-value 0.024), revealing females are more prone to develop the condition. This could mostly be due to estrogens, like pyrogens and medications, which indirectly stimulate the fibrinolytic system leading to the AO by accelerating the clot lysis [28]. The link between the use of contraceptive pills and the development of AO is well explained in the literature [29, 30].

The use of PRF in wound healing and periodontal regeneration adjuvant has yielded promising results. It has been successfully employed in periodontics, oral and maxillofacial surgery, and implant dentistry to treat osseous abnormalities as well as preservation of alveolar ridge [31]. PRF has also demonstrated promising outcomes in the regeneration of the pulp–dentin complex for endodontic operations [32]. However, most of the studies have only shown short-term effects. Further studies are imperative to have a deeper insight into the efficacy and credibility of oral surgical cases and other areas of the body. This can potentiate its use in regular surgical procedures if established as a swift healer.

## 5. Conclusion

PRF showed promising results in the mandibular third molar surgery, thereby decreasing the incidence of AO. The findings suggest that PRF can be used in patients having risk factors for AO. The product has no additives and is autologous thus biocompatible. Its extraction is simple, accessible, and cost-effective. Hence can readily be used in oral surgical procedures for promoting healing in complex invasive procedures involving bone.

## Figures and Tables

**Table 1:** Distribution of patients according to age in both groups.

Age	Group 1		Group 2		Total	
	Frequency	Percentage	Frequency	Percentage	Frequency	Percentage
18–40	40	44.44	48	53.33	88	46.32
41–65	50	55.56	42	46.47	92	53.68
Mean ± SD	42.84 ± 10.52		40.54 ± 9.52		41.35 ± 9.87	

**Table 2:** Distribution of patients according to gender in both groups.

Genders	Group 1		Group 2		Total	
	Frequency	Percentage	Frequency	Percentage	Frequency	Percentage
Male	46	51.11	44	48.89	90	50.0
Female	44	48.89	46	51.11	90	50.0

**Table 3:** Comparison of alveolar osteitis between both groups (*n* = 180).

		Group 1		Group 2	
		Number of patients	Percentage	Number of patients	Percentage
Alveolar osteitis	Yes	02	2.22	11	12.22
	No	88	97.78	79	87.78

*p*-Value is 0.010, which is statistically significant.

**Table 4:** Stratification of alveolar osteitis with respect to age groups.

Age	Group 1		Group 2		*p*-Value
	Alveolar osteitis		Alveolar osteitis		
	Yes	No	Yes	No	
18–40	01	39	05	43	0.142
41–65	01	49	06	36	0.027

**Table 5:** Stratification of alveolar osteitis with respect to gender.

Genders	Group 1		Group 2		*p*-Value
	Alveolar osteitis		Alveolar osteitis		
	Yes	No	Yes	No	
Male	02	44	06	38	0.122
Female	00	44	05	41	0.024

## Data Availability

The data are available with the first author of the manuscript and will be shared on reasonable request.
